# “Cat scratch colon” causing cecal perforation as a rare complication due to barotrauma during colonoscopy: A case report and literature review

**DOI:** 10.1002/deo2.70125

**Published:** 2025-04-29

**Authors:** Hitoshi Hara, Yasuhide Muto, Tomoki Kido, Ryohei Miyata, Moe Tokuda, Tomohiro Maesono, Takahiro Ajihara, Takuma Naritomi, Michio Itabashi

**Affiliations:** ^1^ Department of Surgery Social Welfare Organization Saiseikai Imperial Gift Foundation, Inc. Saiseikai Kazo Hospital Saitama Japan; ^2^ Department of Gastroenterology Social Welfare Organization Saiseikai Imperial Gift Foundation, Inc., Saiseikai Kazo Hospital Saitama Japan

**Keywords:** barotrauma, case report, cat scratch colon, cecal perforation, colonoscopy

## Abstract

“Cat scratch colon” (CSC), characterized by spontaneous bright‐red linear markings or mucosal laceration with bleeding due to air insufflation barotrauma, is a rare complication during colonoscopy. These mucosal lacerations can present as superficial tears that do not generally have clinical repercussions or as deeper tears that damage the muscularis and cause perforation. CSC occurs in the colon with submucosal stiffening disease, such as collagenous colitis; however, in cases unrelated to these diseases, CSC appears in the cecum or ascending colon for anatomical reasons. Herein, we report a case of CSC that caused cecal perforation. A 79‐year‐old woman underwent a colonoscopy for anal bleeding. Although insertion of the colonoscope was easy, as soon as the cecum expanded with air insufflation, the cecal mucosa was torn, and bleeding occurred. The endoscopist determined these findings as shallow mucosal tears and inactive bleeding, and a colonoscopy was completed. She visited our hospital 2 days after colonoscopy with a complaint of abdominal pain that appeared in the morning after colonoscopy. Computed tomography revealed inflammation around the cecum, with free air. Emergency surgery was performed to diagnose an iatrogenic colonic perforation caused by colonoscopy. During surgery, a necrotic area was found in the cecal wall, requiring ileocecal resection. The resected specimen showed cecal mucosal tears with necrosis, which were pathologically consistent with cecal rupture resulting from mucosal laceration. The postoperative course was uneventful. When CSC is encountered along with endoscopic findings of deep mucosal tears in the colon, the possibility of perforation after colonoscopy should be considered.

## INTRODUCTION

“Cat scratch colon” (CSC) is a rare complication during colonoscopy.^1^, ^2^ Excluding cases of colitis with submucosal stiffening, CSCs usually appear in the cecum or ascending colon for anatomical reasons.^1^ The endoscopic gross findings of CSC are characterized by spontaneous mucosal laceration and bleeding caused by barotrauma of air insufflation but not by direct scope trauma.^1^, ^2^ These mucosal lacerations can present as superficial tears that do not generally have clinical repercussions or as deeper tears that can damage the muscularis and cause perforation.^3^ Colonoscopy using carbon dioxide (CO_2_) insufflation is thought to protect against barotrauma injury owing to quicker absorption than ambient air.^4^, ^5^, ^6^ Herein, we report a case of CSC that presented as a rare complication of colonoscopy using CO_2_ insufflation and caused cecal perforation.

## CASE REPORT

**FIGURE 1:**
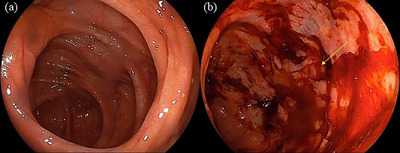
Bleeding is not observed when the colonoscope reaches the cecum (a). As soon as the cecum expanded owing to insufflation to observe its lumen, the cecal mucosa is found to be torn, with fresh bleeding and muscular layer visualization (b: arrow)

**FIGURE 2:**
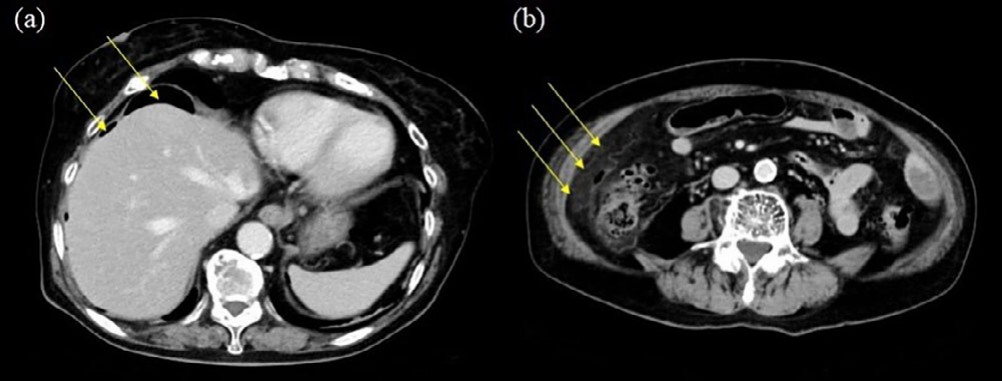
Computed tomography reveals free air on the liver surface (a: arrows) and peri‐cecal inflammation with small air bubbles (b: arrows)

**FIGURE 3:**
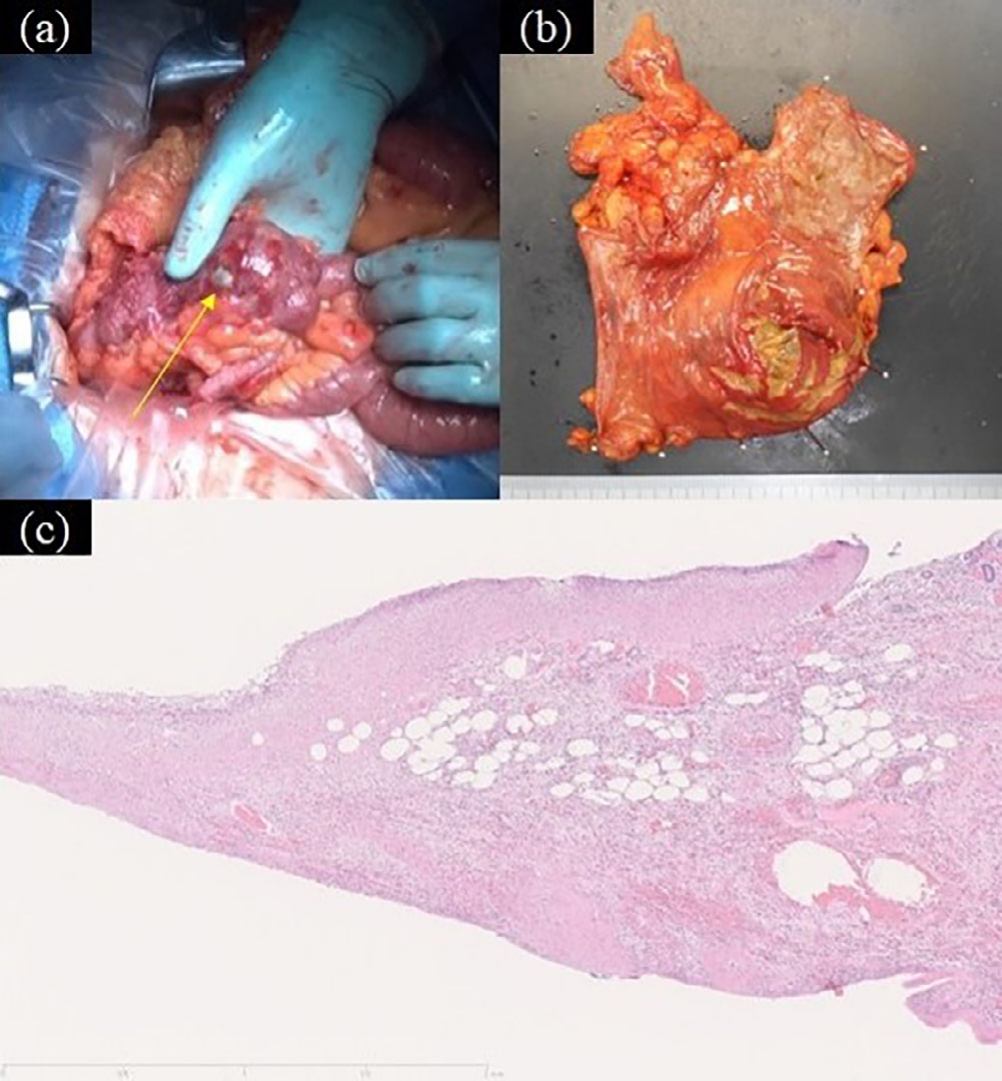
During surgery, a necrotic area measuring 10 mm in diameter is found in the cecal wall (a: arrow), and fecal contamination is not found in the abdominal cavity. The resected specimen shows lacerations of the cecal mucosa, some of which have thinned, ulcerated, and become necrotic (b). Pathological examination shows that some of the lacerated cecal wall has lost the structure of the colon wall and become necrotic, and there is no evidence of colitis such as collagenous colitis (c). These findings are consistent with cecal rupture caused by barotrauma

A 79‐year‐old woman with hypertension underwent a colonoscopy for anal bleeding during bowel movements. All procedures were performed according to our hospital's routine protocol, following bowel preparation with polyethylene glycol, sedation with midazolam, and CO_2_ insufflation. Insertion of the colonoscope was not difficult, and it took approximately 9 min to reach the cecum. Bleeding was not observed when the colonoscope reached the cecum. As soon as the cecum was expanded owing to insufflation to observe its lumen, the cecal mucosa was torn, with fresh bleeding and muscular layer visualization (Figure 1). When tears and cecal bleeding occurred, the tip of the endoscope did not touch the cecal wall directly. These findings were determined as inactive bleeding by the endoscopist, and a colonoscopy was completed. Other endoscopic findings included several ascending colon diverticula, a small polyp in the transverse colon, and internal hemorrhoids. The patient experienced right lower abdominal pain with a fever the morning after colonoscopy and visited our hospital 2 days later. Blood test results revealed high inflammation, with a white blood cell count of 14,700/mm^3^ and a C‐reactive protein level of 19.22 g/dL. Computed tomography revealed inflammation around the cecum, with free air (Figure 2). Emergency surgery was performed to diagnose an iatrogenic colonic perforation caused by colonoscopy. During surgery, a necrotic area measuring 10 mm in diameter was found in the cecal wall, and no fecal contamination was observed in the abdominal cavity. Ileocecal resection was performed. The resected specimen showed lacerations in the cecal mucosa, some of which were thin, ulcerated, and necrotic. Pathological examination showed that some of the lacerated cecal walls had lost the layered structures of the colon wall and become necrotic, with no evidence of colitis such as collagenous colitis (Figure 3). These findings were consistent with the cecal rupture caused by barotrauma. The postoperative course was uneventful, and the patient was discharged on the 10th postoperative day.

## DISCUSSION

The term CSC was first reported in 2007 by McDonnell et al. as bright‐red linear markings in the cecum and ascending colon caused by barotrauma during colonoscopy.^1^ The prevalence of CSC is 0.25%, and patients with CSC tend to be older women or those with a high proportion of collagenous colitis.^1^ In 2013, Baudet et al. reported that 20 cases of CSC were found in 10,715 patients who underwent colonoscopy, 15 of which were patients with diversion colitis and 5 of which were patients with collagenous colitis, and that their occurrence was associated with older age and male sex.^2^ In addition to collagenous colitis and diversion colitis, there have been several reports of CSC associated with ischemic enteritis, Crohn's disease, and chronic colitis due to spirochetosis.^7^, ^8^, ^9^

CSCs associated with some colitis are caused by fibrosis and rigidity of the subepithelium of the colon.^7^ However, in patients without colitis, CSCs are thought to appear specifically in the cecum and ascending colon, which have large diameters for anatomical reasons.^4^ This is because the larger the diameter of the colon, the greater the expansion of the mucosa when the lumen is expanded by air insufflation. This phenomenon is explained by Laplace's law, where the tension in the wall is a product of the intraluminal pressure multiplied by the radius of the lumen.^4^ In other words, in patients with colitis, such as collagenous colitis, in which subepithelial fibrosis occurs in the colon, barotrauma to the stiffened colon results in mild CSC without clinical consequences. Conversely, in patients without such colitis, despite the flexibility of the colonic wall, excessive stretching from air insufflation causes severe CSC in the ascending colon and cecum, which have a large internal diameter.

Air insufflation is unavoidable during colonoscopy for observing the lumen of the colon. CO_2_ insufflation has largely replaced room air insufflation because of its rapid absorption, which reduces pain and flatulence after colonoscopy and enhances patient comfort and recovery.^6^ However, because CSC is a timely complication of colonic distension caused by air insufflation, any kind of gas insufflation cannot completely prevent CSC.

Most reported cases of CSC have only shown endoscopic findings of bright‐red linear markings and are not thought to have clinical repercussions.^1^, ^2^, ^3^ However, there have been few reports of colonic perforation due to CSC. To the best of our knowledge, four cases of CSC causing colonic perforation have been reported.^3^, ^4^, ^5^, ^10^ A summary of five cases, including ours, is shown in Table 1. The median age was 77 years, with 2 cases observed in men and 3 cases in women. Three patients had a history of hypertension. All three patients in whom the insufflation method was described underwent colonoscopy using CO_2_ insufflation. The results of this review are consistent with the inability of CO_2_ insufflation to completely prevent CSC. All CSCs occurred in the right colon, including the ascending colon and cecum. CSCs appeared as endoscopic findings of deep mucosal tears with fresh bleeding and muscular layer visualization in four patients, and three patients required surgery. Pathological findings of non‐specific colitis were observed in one patient, but none of the patients showed findings of collagenous colitis, which has been mostly reported in CSC.

**TABLE 1:** Summary of five cases of “cat scratch colon” causing colonic perforation

Case	Author/year	Age/sex	Past history	Insufflation	Location	CSC findings	Treatment	Colitis findings in pathology
1	Cout WI^10^/2012	87/M	Coronary revascularization surgery, chronic obstructive pulmonary disease	NR	Right colon	Muscularis exposed mucosal lacerations with fresh bleeding	Right hemicolectomy	No (resected specimen)
2	Murphy CJ^4^/2014	77/M	Hypertension, hyperlipidemia	CO_2_	Cecum	Bright‐red liner lesions	Right hemicolectomy	NR
3	Diaz‐Sanchez A^3^/2015	62/F	None	NR	Ascending colon	Muscularis exposed mucosal lacerations with fresh bleeding	Conservative treatment	Non‐specific colitis (Endoscopic biopsy)
4	Barros S^5^/2024	64/F	Hypertension, dyslipidemia	CO_2_	Cecum	Muscularis exposed mucosal lacerations with fresh bleeding	Endoscopic clips for hemostasis and conservative treatment	NR
5	Our case/2025	79/F	Hypertension	CO_2_	Cecum	Muscularis exposed mucosal lacerations with fresh bleeding	Ileocecal resection	No (resected specimen)

Abbreviations: CO_2_, carbon dioxide; CSC, cat scratch colon; F, female; M, male; NR, not reported.

In conclusion, it is not possible to completely prevent CSC, even in colonoscopy with CO_2_ insufflation, because the characteristics of CSCs appear in a timely manner owing to expansion with insufflation. Although most CSCs do not generally have clinical repercussions when encountering CSC with endoscopic findings of deep mucosal tears in the right colon, where the muscle layer is exposed and fresh bleeding is observed, the possibility of perforation after colonoscopy should be considered. When endoscopists encounter such findings, it is recommended to hospitalize the patient after the endoscopy and monitor them closely while fasting, allowing for a prompt response in the event of colonic perforation.

## CONFLICT OF INTEREST STATEMENT

None.

## ETHICS STATEMENT

Approval of the research protocol by an Institutional Reviewer Board: N/A. This study has been performed in accordance with the tenets of the Declaration of Helsinki. The patient's anonymity was maintained.

## INFORMED CONSENT

Written informed consent was obtained from the patient for publication of this report.

## CLINICAL TRIAL REGISTRATION

N/A.
